# Risk of Healthcare Worker Burnout in Africa during the COVID-19 Pandemic

**DOI:** 10.5334/aogh.3150

**Published:** 2021-01-05

**Authors:** Jose D. Debes, Nasreen S. Quadri, Amir Sultan, Mirghani Yousif, Sophia Ibrahim Ali, Johnstone Kayandabila, Ifeorah Ijeoma, Kenneth Ssebambulidde, Lucy Ochola, Abdelmajeed Moussa

**Affiliations:** 1 Department of Medicine & School of Public Health, University of Minnesota, Minneapolis, MN, USA; 2 Department of Medicine, University of Minnesota, Minneapolis, MN, USA; 3 Department of Gastroenterology, Addis Ababa University, Addis Ababa, Ethiopia; 4 School of Pharmacy, University of Gezira, Gezira, Sudan; 5 School of Public Health, University of Minnesota, Minneapolis, MN, USA; 6 Department of Medicine, Arusha Lutheran Medical Centre, Arusha, Tanzania; 7 Department of Virology, University of Nigeria, Nsukka, Nigeria; 8 Department of Medicine, Makerere University, Kampala, Uganda; 9 Institute for Primate Research, Nairobi, Kenya; 10 Department of Gastroenterology, Aswan University Hospital, Aswan, Egypt

## Abstract

COVID-19 is now impacting every country in Africa and healthcare workers (HCWs) across the continent remain susceptible to professional burnout. We designed a 43-question survey addressing multiple aspects of the COVID-19 pandemic. The survey was anonymous, distributed via email and phone messaging to 13 countries in Africa. We obtained 489 analyzable responses. 49% off HCWs reported a decrease in income, with the majority experiencing between 1–25% salary reduction. Sixty-six percent reported some access to personal protective equipment (PPE), 20% had no access to PPE and only 14% reported proper access. Strikingly, the percentage reporting never feeling depressed changed from 61% before the pandemic to 31% during the pandemic, with an increase in daily depression from 2% to 20%. We found no association between depression and change in income, household size, availability of PPE or lockdown. Safety concerns related to stigma from being HCWs affected 56% of respondents.

To the Editor,

COVID-19 is now impacting every country in Africa [1]. Public health authorities in the continent have responded strongly with the creation of an African taskforce for the novel coronavirus and a multifaceted funding approach to the pandemic [2]. Despite this, healthcare workers (HCWs) across the continent remain susceptible to a variable degree of institutional support that places them at high risk for personal burden and professional burnout [3,4]. We designed and implemented a 43-question survey addressing personal and medical perceptions of HCWs during the pandemic. The survey was anonymous and distributed via email and phone messaging to 13 countries in Africa through the African Hepatitis B Network (africanhepbnetwork.org) from April to May 2020. We used risk ratio analysis to quantify the relationship between binary variables and Chi-square testing to quantify the statistical significance of these relationships. Tables (2 × 2) were constructed for survey questions of interest and the proportion (“risk”) of a particular survey response (out of all the responses) was calculated using these tables. Chi-square tests using a standard p-value of 0.05 were run using SAS. The study was approved by the ethics committee of Hennepin Healthcare. We obtained 489 analyzable responses out of a total of 535. Participants from six countries (Ethiopia, Tanzania, Nigeria, Egypt, Uganda, Sudan) represented over 90% of our survey data, which limits generalizability across the continent. Remaining data included Kenya, Sierra Leone, Somalia, The Gambia, Rwanda, South Sudan, and Malawi. The median age of respondents was 30 years (IQR 26–36) and 73% identified as male. Most HCWs included in survey data were physicians (62%), followed by medical/clinical officers (8%), nurses (7%), students (6%) and pharmacists (6%). Seventy-two percent reported living under stay-at-home orders with some intercountry variation. Questions to address depression utilized the framework from the Patient Health Questionnaire (PHQ-2) asking survey respondents to answer questions about depressive symptoms currently and prior to the pandemic, which had a risk of availability bias. Strikingly, the percentage of HCWs reporting never feeling depressed was 61% prior to the pandemic on retrospective questioning as compared to 31% during the pandemic at the time of survey completion. Similarly, a higher percentage of respondents asserted daily depression symptoms during the pandemic (20%) in comparison to prior to the pandemic (2%). We found no association between self-reported depressive symptoms during the pandemic and change in income, household size, availability of PPE or lockdown as noted in Table 1. We found an inverse association between self-reported depression and change in workload (RR 0.86, CI 0.76–0.96, p = 0.01). Safety concerns related to stigma from being HCWs affected 56% of respondents. Fears centered on risk of infection due to lack of resources (33%), risk of infection due to community transmission (23%), economic insecurity (11%), and social stigma (11%). Our survey identifies significant concerns related to personal safety, access to PPE, and social stigma among HCWs throughout Africa. Limitations include snowball sampling, distribution in English language only, availability bias of respondents in answering questions about the past and underrepresentation of all respondents for each question (survey respondents were given the option to leave a question response blank if preferred). Moreover, survey was at one timepoint, hence responses from pre-pandemic period would be considered retrospective. HCWs are faced with a double burden of societal effects of the pandemic’s mitigation strategies like lockdowns as well as work challenges and subsequent mental health effects of working on the frontlines in healthcare during the pandemic. Institutions in the region should invest in supporting HCWs during the COVID-19 pandemic with both tangible resources to sustain the medical work and address mental health burden of the pandemic. The skills and resilience of HCWs will define medical care in the region for years to come. Full details of the survey tool and resultant data are currently pending publication.

**Table 1:** Association between risk variables and perceptions by healthcare workers.


SELF-REPORTED DEPRESSION OR HOPELESSNESS		

VARIABLE	RISK RATIO (95% CI)	P-VALUE

Household size > 4	1.01 (0.89, 1.14)	0.889

Lockdown recommendation	0.94 (0.82, 1.07)	0.3388

Change in work frequency	0.86 (0.76, 0.96)	0.0172

Decrease in income	1.08 (0.96, 1.22)	0.2289

Any access to PPE	0.95 (0.84, 1.08)	0.4275

**FEAR FOR PERSONAL SAFETY DUE TO COVID-19 LOCKDOWN**		

**VARIABLE**	**RISK RATIO (95% CI)**	**P-VALUE**

Household size > 4	1.16 (1.01, 1.34)	0.0432

Any access to PPE	0.85 (0.73, 1.00)	0.0691

**CONCERNS OF EXPOSING FAMILY TO THE VIRUS**		

**VARIABLE**	**RISK RATIO (95% CI)**	**P-VALUE**

Household Size > 4	0.99 (0.93, 1.06)	0.8555

Access to PPE	1.05 (0.96, 1.14)	0.2372

**SUSPECTED SELF-EXPOSURE TO THE VIRUS**		

**VARIABLE**	**RISK RATIO (95% CI)**	**P-VALUE**

Doctors	1.82 (1.41, 2.37)	<0.0001

Nurses	0.65 (0.37, 1.15)	0.10

Access to PPE	0.81 (0.64, 1.03)	0.0997

